# Big data analysis and machine learning of the role of cuproptosis-related long non-coding RNAs (CuLncs) in the prognosis and immune landscape of ovarian cancer

**DOI:** 10.3389/fimmu.2025.1555782

**Published:** 2025-02-25

**Authors:** Mingqin Kuang, Yueyang Liu, Hongxi Chen, Guandi Chen, Tian Gao, Keli You

**Affiliations:** ^1^ Gynecology and Oncology Department of Ganzhou Cancer Hospital, Ganzhou, Jiangxi, China; ^2^ Department of Gynecology, Guangdong Provincial People’s Hospital (Guangdong Academy of Medical Sciences), Southern Medical University, Guangzhou, China

**Keywords:** ovarian cancer, cuproptosis, long non-coding RNAs, prognosis, immune landscape

## Abstract

**Background:**

Ovarian cancer (OC) is a severe malignant tumor with a significant threat to women’s health, characterized by a high mortality rate and poor prognosis despite conventional treatments such as cytoreductive surgery and platinum-based chemotherapy. Cuproptosis, a novel form of cell death triggered by copper ion accumulation, has shown potential in cancer therapy, particularly through the involvement of CuLncs. This study aims to identify risk signatures associated with CuLncs in OC, construct a prognostic model, and explore potential therapeutic drugs and the impact of CuLncs on OC cell behavior.

**Methods:**

We analyzed ovarian cancer data (TCGA-OV) from the TCGA database, including transcriptomic and clinical data from 376 patients. Using Pearson correlation and LASSO regression, we identified 8 prognostic CuLncs to construct a risk signature model. Patients were categorized into high- and low-risk groups based on their risk scores. We performed survival analysis, model validation, drug sensitivity analysis, and *in vitro* experiments to assess the model’s performance and the functional impact of key CuLncs on OC cell proliferation, invasion, and migration.

**Results:**

The prognostic model demonstrated significant predictive power, with an area under the curve (AUC) of 0.702 for 1-year, 0.640 for 3-year, and 0.618 for 5-year survival, outperforming clinical pathological features such as stage and grade. High-risk OC patients exhibited higher Tumor Immune Dysfunction and Exclusion (TIDE) scores, indicating stronger immune evasion ability. Drugs such as JQ12, PD-0325901, and sorafenib showed reduced IC50 values in the high-risk group, suggesting potential therapeutic benefits. *In vitro* experiments revealed that knockdown of LINC01956, a key CuLnc in the risk signature, significantly inhibited the proliferation, invasion, and migration of OC cells (P<0.05).

**Conclusion:**

Our study identified a prognostic risk model based on CuLncs and explored their potential as therapeutic targets in OC. The findings highlight the importance of CuLncs in OC prognosis and immune response, providing new insights for future research and clinical applications.

## Introduction

Ovarian cancer(OC) is a severe malignant tumor that poses a significant threat to the life and health of women (1–4). According to statistical data released by the American Cancer Society, it is projected that in 2024, the United States will diagnose 2,001,140 new cases of ovarian cancer in women, with an estimated 611,720 mortality cases in the same year, and a five-year survival rate of approximately 50% (5). The traditional treatment methods for OC mainly include cytoreductive surgery and chemotherapy regimens based on platinum drugs, yet the prognosis for patients remains grim (6–9). This stark clinical reality underscores the urgent need for preclinical models (10, 11). Models constructed from data extracted from actual ovarian cancer cases in the TCGA database can provide scientific tools for the design of new treatment strategies and the exploration of potential targets.

Cuproptosis is a recently discovered way of cells death (12). It happens when copper ions pile up inside cells. This leads to a clumping of certain proteins in the mitochondria—specifically, the acylated lipoproteins. Additionally, it causes a shaking up of iron-sulfur cluster proteins. All of this culminates in cell death (13). Since the concept of cuproptosis was introduced, numerous researchers and research institutions have been committed to exploring its application in cancer therapy and have achieved significant progress, especially in the field of cuproptosis-related long non-coding RNAs (CuLncs) (14–16). Studies have shown that the downregulation of lncRNAs such as WARS2-AS1 and MKLN1-AS can enhance the sensitivity of cells to elesclomol-induced cuproptosis (17). Additionally, miR-21-5p enhances the proliferation and invasiveness of tumor cells through the modulation of FDX1 expression. And FDX1 is a factor linked to unfavorable outcomes in individuals diagnosed with clear cell renal cell carcinoma. Subsequent *in vitro* studies have demonstrated that the downregulation of miR-21-5p markedly impedes the oncogenic expansion and metastatic potential of renal carcinoma cell lines ACHN and OSRC-2 (18). Although there are studies on CuLncs in the field of OC, most research focuses on constructing prognostic models based on the expression levels of CuLncs, rather than validating specific lncRNAs at the cellular level (19–22). In addition, there is a close relationship between CuLncs and the cancer immune microenvironment, which is evident in various types of cancer, especially in triple-negative breast cancer (TNBC). Studies have shown that in TNBC, CuLncs also demonstrate significant prognostic value and the ability to regulate the immune microenvironment. One such study identified 111 lncRNAs associated with cuproptosis through co-expression analysis and constructed a prognostic model that included CuLncs such as MELTF-AS1. The results showed that in the high-risk group, there was an increased infiltration of immunosuppressive cells in the tumor tissue, while patients in the low-risk group had significantly prolonged survival times (23). This indicates that CuLncs can not only predict the prognosis of TNBC patients but also influence tumor progression by modulating the immune microenvironment. The potential applications of CuLncs in immunotherapy have also attracted attention. For example, certain CuLncs may affect the response to immunotherapy by regulating the expression of immune checkpoint genes (24). Therefore, investigating the prognostic and immune landscape associations between CuLncs and ovarian cancer is of great significance for improving the prognosis of OC patients.

In this study, we aim to identify risk signatures by analyzing the expression differences of CuLncs in OC. And constructing and validating a prognostic model for OC patient survival outcomes by using machine learning analysis methods. Additionally, this study explores potential therapeutic drugs for patients in different prognostic groups and ultimately verifies the impact of key CuLncs on the proliferation, invasion, and migration capabilities of OC cells through a series of *in vitro* cellular experiments.

## Materials and methods

### Study design

**Figure 1:**
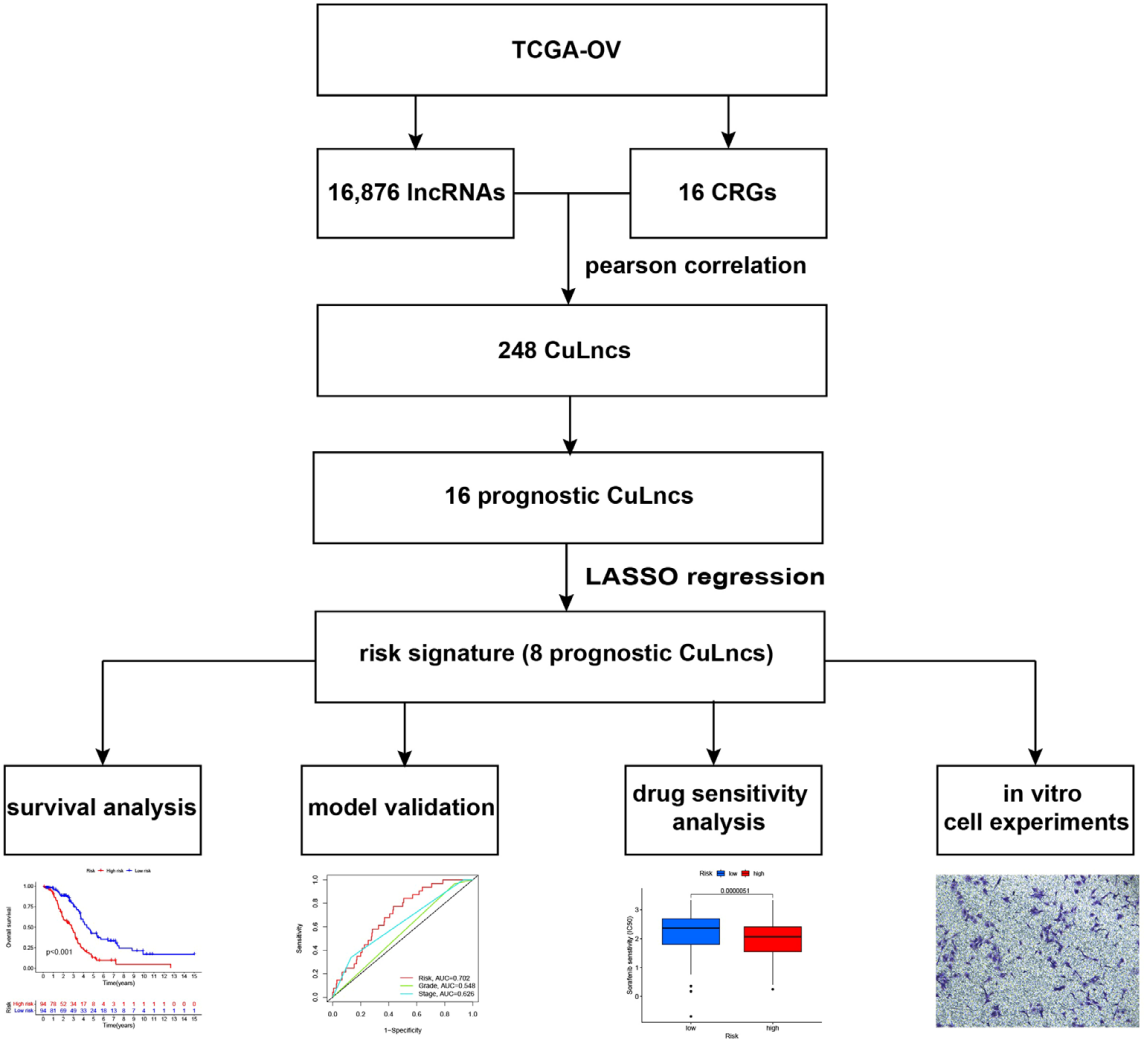
Study flow chart. TCGA-OV, Ovarian cancer data of The Cancer Genome Atlas database. lncRNAs, long noncoding RNAs. CRGs cuproptosis-related genes. CuLncs, cuproptosis-related lncRNAs.

**Figure 1** delineates the overall design of this study (**Figure 1**). Ovarian cancer data (TCGA-OV) were downloaded from TCGA (the cancer genome atlas), encompassing transcriptomic and clinical data from 439 ovarian cancer patients. To enhance the robustness of the prognostic model, patients lacking survival data were excluded (n=63), resulting in the inclusion of data from 376 ovarian cancer patients for analysis. 8 prognostic CuLncs were identified as risk signatures through Pearson correlation analysis and LASSO regression. Subsequently, a predictive model was formulated utilizing this risk signatures, enabling the categorization of participants into distinct groups characterized by high and low risk. Survival analysis, model validation, gene set enrichment analysis, and drug sensitivity analysis were performed within these risk groups. Furthermore, the impact of key CuLncs on the proliferative, migratory, and invasive capabilities of OC cells was further validated *in vitro*.

### Defining CuLncs and building a prognostic model

The transcriptomic data of the 376 ovarian cancer patients included in the study were annotated based on GENCODE Release 29 (GRCh38.p12), resulting in 16,876 lncRNAs. Concurrently, 16 copper death-related genes (CRGs) were identified through literature review, including ATP7A (25), PDHB (26), DLAT (27), LIPT2 (28), DBT (29), NFE2L2 (30, 31), NLRP3 (32), CDKN2A (33), DLST (34), LIAS (35), FDX1 (36, 37), DLD (38), PDHA1 (39), GCSH (40), SLC31A1 (41), and MTF1 (42). In R Studio, relevant R packages such as limma, dplyr, ggalluvial, and ggplot2 were loaded. Employing a threshold of a Pearson correlation coefficient>0.4 and *P*-value< 0.001, an analysis was performed to correlate the aforementioned lncRNAs with CRGs, thereby pinpointing CuLncs. Subsequently, the identified CuLncs data were subjected to univariate regression analysis with the survival time data from TCGA-OV to determine the prognostic CuLncs associated with ovarian cancer survival time. Finally, a risk signature composed of 8 prognostic CuLncs was selected through LASSO regression analysis and cross-validation to construct the prognostic model. For each OC patient, a prognostic score was derived from the expression levels of the CuLncs within the risk signature, along with their respective coefficients, according to the subsequent equation:


$$\text{Risk score=}\sum_{\text{i}}^{n}\text{coef}\left(\text{i}\right)\times \text{Exp(i)}$$

Subsequently, individual prognostic risk scores were determined for each patient employing the designated risk computation formula, with the median score serving as the demarcation point to segregate patients into high- and low-risk prognostic categories.

### Survival analysis

Initially, the clinical data of 376 OC patients were organized, including their age, International Federation of Gynecology and Obstetrics (FIGO) stage, and grade. Subsequently, in R Studio, the caret package was loaded to randomly divide the 376 ovarian cancer patients into the training set (n=188) and the test set (n=188), followed by a chi-square test. Then, prognostic risk scoring and survival analysis were performed for both the training and validation sets. Utilizing the risk scores obtained from the signature, patients with OC were categorized into high- and low-risk groups. Ultimately, the survminer package was deployed to generate the risk score curves, survival status scatter plots, and Kaplan-Meier survival curves for both the training and test sets.

### Validation of prognostic model performance

The prognostic risk score’s association and independence were examined through univariate and multivariate regression analyses. Subsequently, to validate the prognostic model’s capabilities in this study, time-dependent ROC curves were employed to assess its predictive power at 1, 3, and 5 years. Additionally, the discriminative ability of the prognostic risk score was compared with stage and grade using ROC curves. Furthermore, a concordance index test was conducted. A nomogram was constructed based on the age of OC patients, FIGO stage, grade, and prognostic risk score, and the calibration of this nomogram was evaluated using calibration curves.

### Drug sensitivity analysis

In R studio, the random seed was set to 12345, and necessary packages were loaded, including the limma package, ggpubr package, pRRophetic package, and ggplot2 package. All gene expression data files for TCGA-OV, as well as the prognostic CuLncs data for the training and validation sets, were read. Various drug information from the “pRRophetic” package and risk scores for each sample were extracted. A loop was conducted to screen the 50% inhibiting concentration (IC50) for each drug for every sample, using *P*<0.001 as the filtering criterion for drug sensitivity. The drug sensitivity results were then merged with the risk score results into a new data matrix. The ggboxplot function was employed to ascertain the disparities in drug responsiveness between the high- and low-risk prognostic groups for each pharmaceutical agent, subsequently generating the corresponding boxplots.

### Prognostic analysis of high and low expression of CuLncs in the risk signature

The expression levels of CuLncs in the risk signature and the survival data of OC patients, including survival time and status, were extracted from TCGA-OV. Subsequently, patients were categorized into high and low expression groups based on the median expression level of CuLncs within the risk signature. Kaplan-Meier survival curves were plotted using the survminer package.

### Cell culture

Human ovarian cancer cell lines A2780 and SKOV3, sourced from the Chinese Academy of Sciences (Shanghai) Cell Bank, are propagated in Dulbecco’s Modified Eagle Medium with high glucose (HyClone), supplemented with 10% fetal bovine serum (Thermo). These cells are nurtured in a humidified chamber at a temperature of 37°C and a CO2 concentration of 5%.

### Real-time quantitative PCR (RT-qPCR)

Total RNA was extracted from A2780 and SKOV3 cells 48 hours post-siRNA transfection using the RNeasy Mini Kit (Qiagen). RNA quantity and purity were assessed using a NanoDrop 2000 spectrophotometer (Thermo Fisher Scientific), ensuring an A260/A280 ratio of 1.8–2.0. First-strand cDNA was synthesized from 1 μg of RNA using the High-Capacity cDNA Reverse Transcription Kit (Applied Biosystems) with the following conditions: 25°C for 10 min, 37°C for 120 min, and 85°C for 5 min. Primers for LINC01956 and GAPDH were designed using Primer3 or Primer-BLAST, and RT-qPCR was performed using PowerUp SYBR Green Master Mix (Thermo Fisher Scientific) on a StepOnePlus Real-Time PCR System (Applied Biosystems). Each 20 μl reaction contained 10 μl of master mix, 1 μl of each primer (10 μM), and 2 μl of cDNA template. Cycling conditions were 95°C for 10 min, followed by 40 cycles of 95°C for 15 s and 60°C for 1 min. Relative expression levels of LINC01956 were calculated using the 2^-ΔΔCt method, normalized to GAPDH.

### Screening siRNA

Initially, A2780 and SKOV3 cells were cultured to reach 70-80% confluence. Subsequently, 3 siRNAs (siRNA-1, siRNA-2, siRNA-3) were transfected into the cells using a transfection reagent, with triplicates for each siRNA. A control group consisting of cells without siRNA transfection was also included. After a 48-hour post-transfection period, the cells were collected for real-time quantitative PCR (qPCR) to assess the levels of LINC01956.

### Cell Counting Kit-8 Assay

Using M5 HiPer Cell Counting Kit (CCK) (MF128-02, polymery) Bio-Tek Enzyme-Linked Immunosorbent Assay (ELISA) Reader ELX800. After trypsinization and counting, cells were adjusted to a concentration of approximately 30,000 cells/ml. A2780 and SKOV3 cells were seeded into a 96-well plate at 100 μl of cell suspension per well, containing about 3,000 cells per well, with triplicates for each sample. The CCK-8 assay reagent was introduced into each well for a 4-hour incubation period. Optical density at 450 nm was quantified utilizing a microplate spectrophotometer at time points of 0, 24, 72, and 96 hours post-seeding.

### Transwell assay for cell invasion

The SKOV3-NC group, SKOV3 treated group, A2780-NC group, and A2780 treated group were subjected to the Transwell assay to evaluate their invasive capabilities. A total of 3,000 cells per cohort were inoculated into the apical chamber of the Transwell inserts, while the basal chamber was filled with RPMI-1640 medium enriched with FBS. After a 48-hour cultivation period, cells adhering to the upper side of the membrane were carefully eradicated with a cotton swab, followed by the fixation and staining of the cells that had traversed to the lower membrane surface using crystal violet. Once dried, the stained cells were examined under a light microscope. Using Mingmei Microscopic Digital Imaging System (Mingmei) and 6-well Plate (Corning).

### Scratch test for assessing cell migration

Cells from each experimental group, during their logarithmic growth phase, were resuspended in DMEM medium supplemented with 10% FBS and seeded into a 6-well plate. Upon achieving approximately 80% confluence, a linear incision was created at the well’s base using the tip of a 20 μL sterile pipette. Subsequently, the wells were thoroughly rinsed twice with PBS to remove debris, and then treated with mitomycin C (20 μg/mL) in FBS-free medium to inhibit cell proliferation and its influence on the experimental results. Images documenting the wound closure were captured at 0 and 24 hours post-injury. The area of the scratch was quantified using an inverted microscope, with S0 denoting the initial incision and S1 indicating the area 24 hours post-wounding. The cell migration ability was calculated using the formula: Migration rate = ((S1 - S0)/S0) × 100%.

Using 8.0 μ m Transwell chamber (Corning), 24 well plate (Corning), cotton swab, fully cultured, serum-free medium (Gibco), trypsin (Gibco), PBS (Gibco), Matrigel (Corning), 4% paraformaldehyde (Zhongshan Jinqiao), crystal violet staining solution (Jinclone).

### Statistical analysis

The experimental data are depicted as the mean ± standard deviation and were subjected to analysis using SPSS 21.0 software. For assessing differences in quantitative data between two groups, a two-sample t-test was applied; whereas for evaluating quantitative data across three or more groups, a one-way ANOVA was employed. Graphical representations and charts were crafted using GraphPad Prism (version 9.5) and the R programming language (version 4.1.3). Throughout this study, each experiment was performed with a minimum of three biological replicates, and statistical significance was determined at a *P*-value threshold of less than 0.05.

## Results

### Identification of prognostic CuLncs in ovarian cancer

We randomly divided 376 OC patients from TCGA-OV into training and test sets, as shown in **Supplementary Table 1**, with no clinical characteristics differences between the two datasets, indicating that the study was conducted with random and rational grouping (**Supplementary Table 1**). As depicted in **Figure 1**, we obtained 16,876 lncRNAs from TCGA-OV and identified 248 CuLncs through Pearson correlation analysis with 16 CRGs. This study utilized a Sankey diagram to illustrate the relationship between the 248 CuLncs and 16 CRGs (**Supplementary Figure 1A**). Subsequently, a COX correlation analysis was conducted between the 248 CuLncs and the survival time of OC patients, identifying 16 prognostic-related CuLncs with a *p*-value cutoff of less than 0.05, and their hazard ratios (HRs) and values were displayed using a forest plot (**Supplementary Figure 1B**). Notably, LINC01956 had an HR and 95% CI of 3.028 (1.098-8.352), suggesting that LINC01956 could be a contributory factor linked to unfavorable outcomes in OC patients.

### Constructing and validating the prognostic risk model

**Figure 2:**
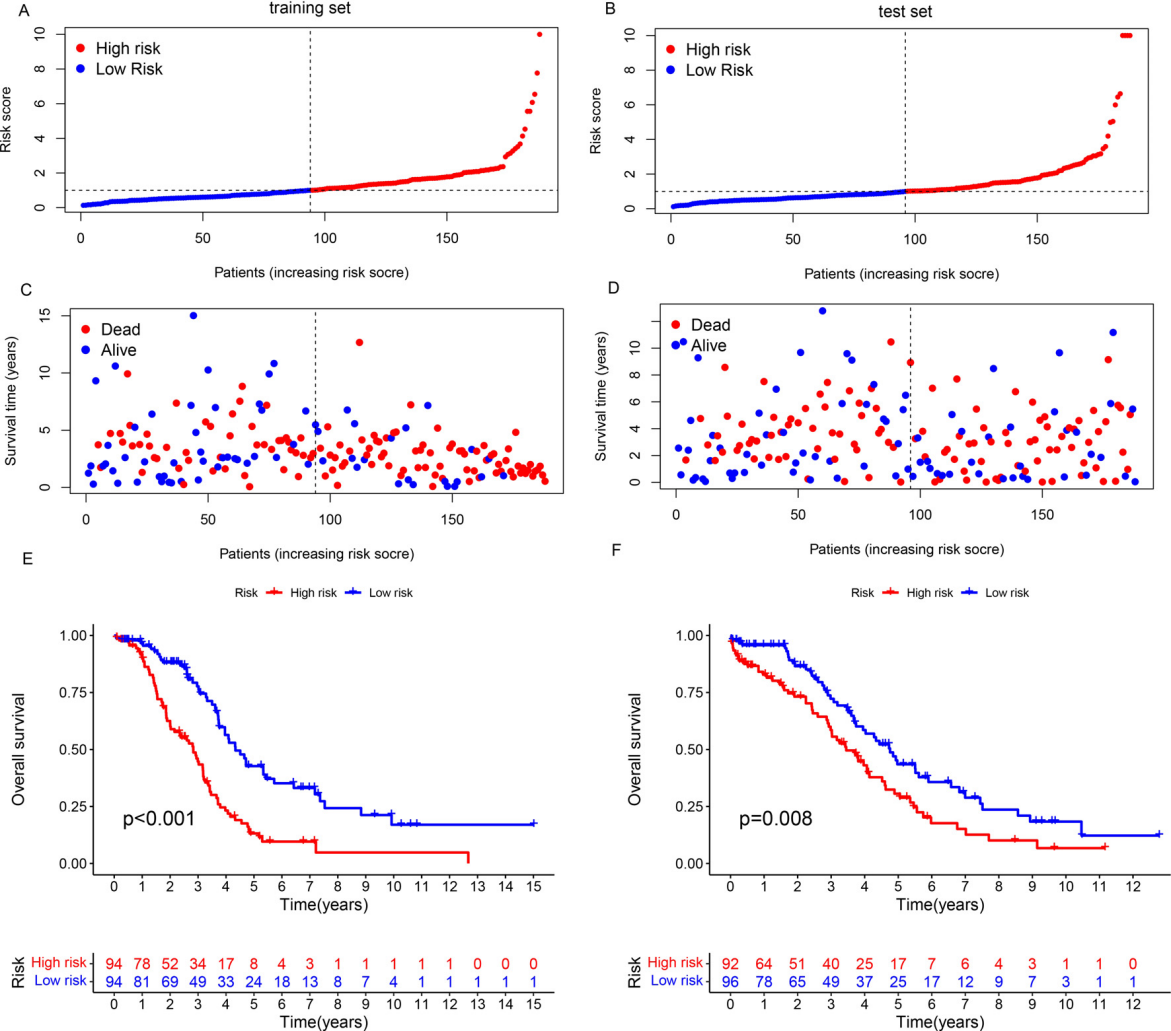
Development of the CuLncs prognostic signature for OC. The risk score of prognostic signature **(A, B)** and survival status **(C, D)** in the training set and test set. Kaplan−Meier survival curves of OS [**(E)** *P*<0.001, **(F)** *P*=0.008] in the high- and low-risk groups of OC patients in the training set and test set.

Utilizing the 16 CuLncs associated with prognosis, we subsequently implemented Lasso Cox regression analysis coupled with 10-fold cross-validation within the training set to ascertain risk scores for the development of the prognostic model (**Supplementary Figures 1C, D**). The prognostic risk score for each participant was derived from the coefficient values associated with the risk signatures outlined in the supplementary material (**Supplementary Table 2**), with stratification into high- and low-risk groups predicated on the median risk score. Survival analysis in the training set indicated that as the risk score increased, the survival time of OC patients decreased, with the high-risk group having a significantly shorter survival time (**Figures 2A, C, E** *P*<0.001). Similarly, a similar conclusion was reached in the test set (**Figures 2B, D, F** *P*=0.008). This suggests that the risk score can not only act as a predictive marker for the survival duration of OC patients but also help identify patient groups with different survival probabilities.

### Testing the prognostic model’s performance

**Figure 3:**
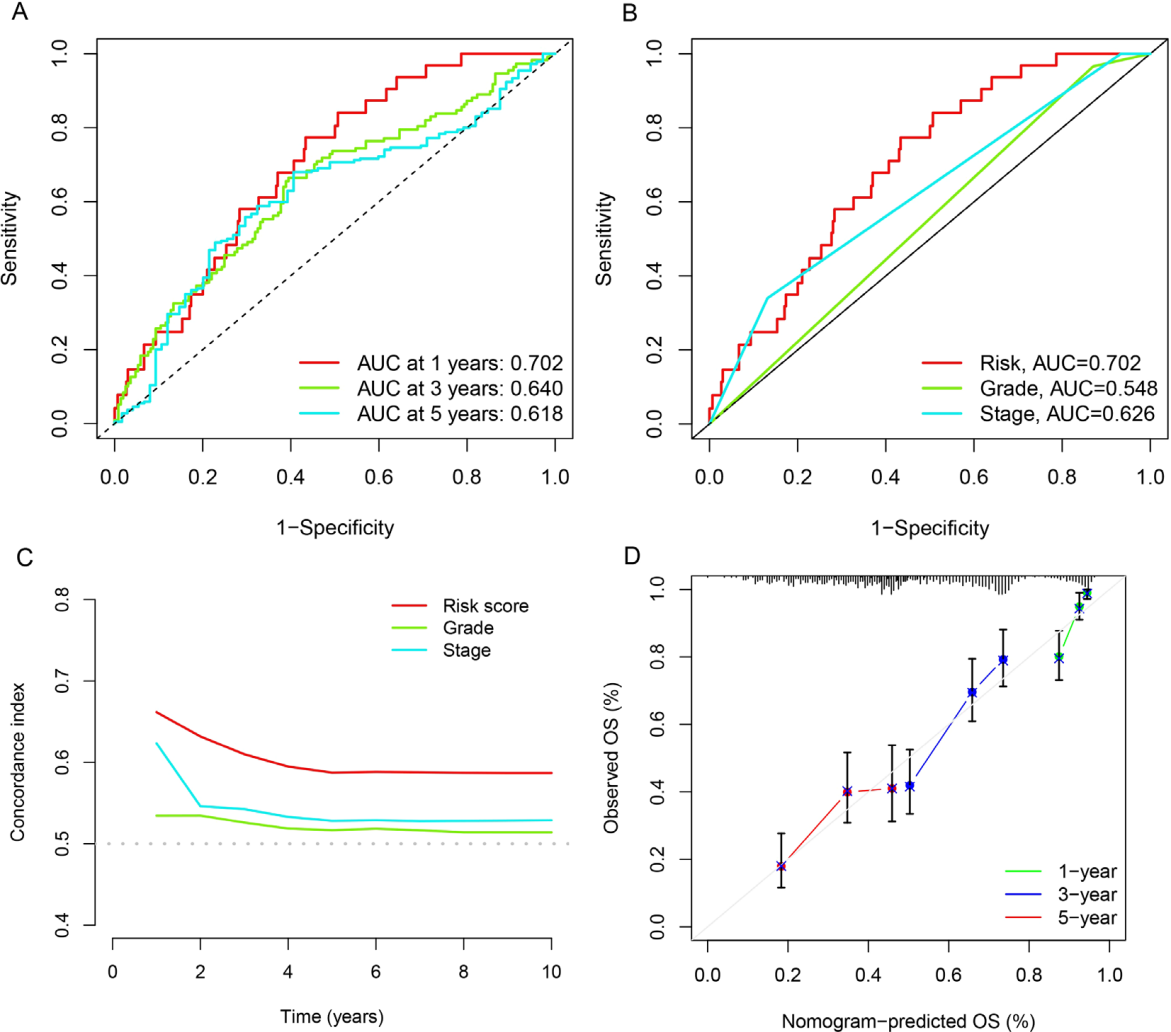
Evaluation of the accuracy of the CuLncs prognostic signature in OC. ROC curve of the model for predicting 1-year, 3 year, and 5-year OS **(A)**. ROC curve of the model considering the risk score and clinical characteristics **(B)**. C-index curve of the prognostic signature **(C)**. Calibration curves for nomogram **(D)**.

The ROC curve and concordance index analysis were utilized to assess the discriminative power of the prognostic model, revealing the area under the curve (AUC) to be 0.702 for 1-year, 0.640 for 3-year, and 0.618 for 5-year survival (**Figure 3A**). Further analysis indicated that compared to clinical pathological features such as stage and grade, the risk score model demonstrated stronger predictive power (AUC=0.702, **Figure 3B**). Similar conclusions were drawn from the concordance curves (**Figure 3C**). Furthermore, both univariate and multivariate regression analyses were applied to this prognostic model, establishing the risk score as an independent predictor of OC patient outcomes. (**Supplementary Figures 2A, B**). This study also constructed a nomogram based on the age, stage, grade, and risk score of OC patients (**Supplementary Figure 2C**). Calibration curves indicated that the nomogram was fairly accurate in predicting the 1-year, 3-year, and 5-year survival times of OC patients (**Figure 3D**).

### JQ12, PD-0325901, and sorafenib may be potential therapeutic agents for high-risk OC patients

**Figure 4:**
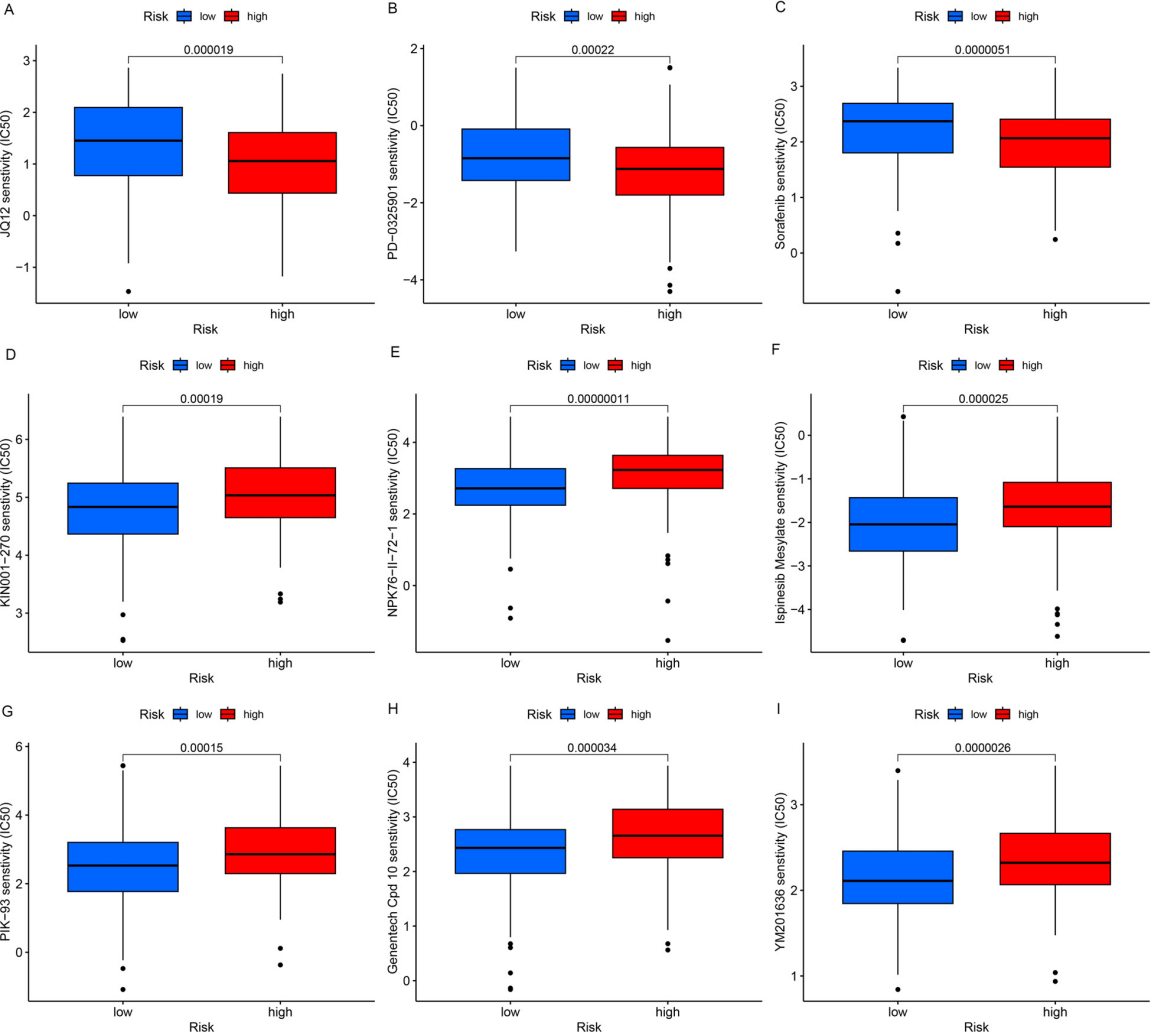
Drug sensitivity analysis. OC patients with a high risk score had a higher IC50 value for many therapeutic drugs than patients with a low risk score **(A–I)**.

**Figure 4** illustrated the IC50 values of different drugs for both high and low-risk groups. Notably, the drugs JQ12 (**Figure 4A**, P=0.000019), PD-0325901 (**Figure 4B**, P=0.00022), and sorafenib (**Figure 4C**, P=0.0000051) demonstrated reduced IC50 values within the high-risk cohort, implying that patients in this group, who have a pessimistic prognosis, might derive therapeutic advantages from such pharmaceuticals. However, further relevant trials are needed to validate these findings.

### Immune landscape of OC patients in high and low risk groups

This study evaluated the differences in Tumor Immune Dysfunction and Exclusion (TIDE) scores between high-risk and low-risk groups of OC patients. As shown in the figure, the high-risk OC group exhibited higher TIDE scores, indicating a stronger ability to evade the immune system. This suggests that they may be less responsive to immunosuppressive drug treatments (**Supplementary Figure 3A**, p<0.05). Additionally, this study investigated the differences in immune activity between the high-risk and low-risk populations. The results revealed significant differences in immune activity among these groups (**Supplementary Figure 3B**). Specifically, the high-risk group showed higher levels of gene sets associated with MHC I expression (P<0.001), Type I interferon (INF) response, pro-inflammatory activity, and cytolytic activity (P<0.01) compared to Type II INF response, T cell co-inhibition, and APC co-inhibition (P<0.05). Notably, the expression of Type I interferon was significantly lower in the high-risk group compared to the low-risk group.

### Elevated LINC01956 expression correlates with adverse outcomes in individuals with OC

**Figure 5:**
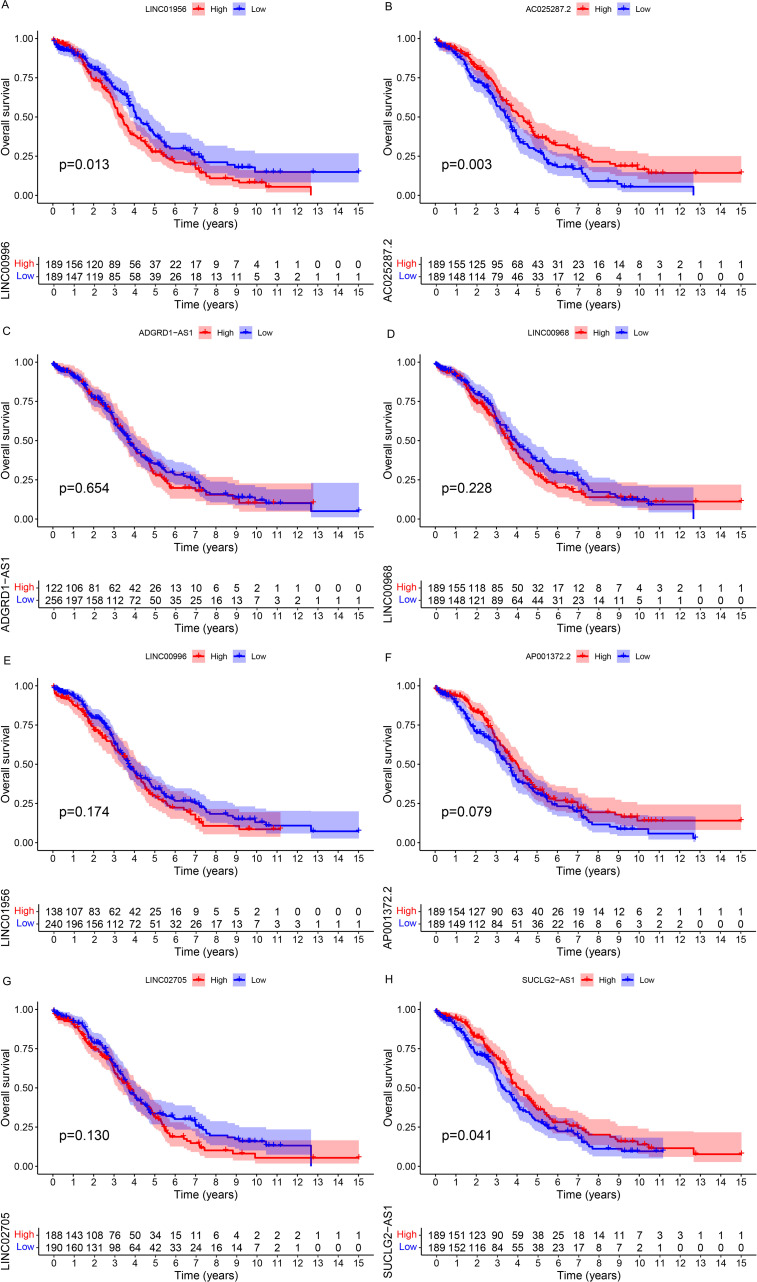
Survival analysis. The Kaplan-Meier survival curves for each CuLnc in the risk signature **(A-H)**.

To further determine which CuLncs in the risk signature play a key role in leading to poor prognosis in OC patients, we conducted survival analyses for each of the 8 CuLncs in the risk signature (**Figure 5**). The results showed that OC patients with high expression of LINC01956 had a worse prognosis (**Figure 5A**, *P*=0.013), which corroborates the strong risk factor for OC prognosis mentioned earlier in **Supplementary Figure 1B**. In contrast, high expression of AC025287.2 and SUCLG2-AS1 were protective factors for OC prognosis (**Figure 5B**, P=0.003, **Figure 5H**, *P*=0.041). Therefore, LINC01956 was selected as the subject for subsequent experimental research.

### Knockdown of LINC01956 inhibits the proliferation of A2780 and SKOV3 cells

**Figure 6:**
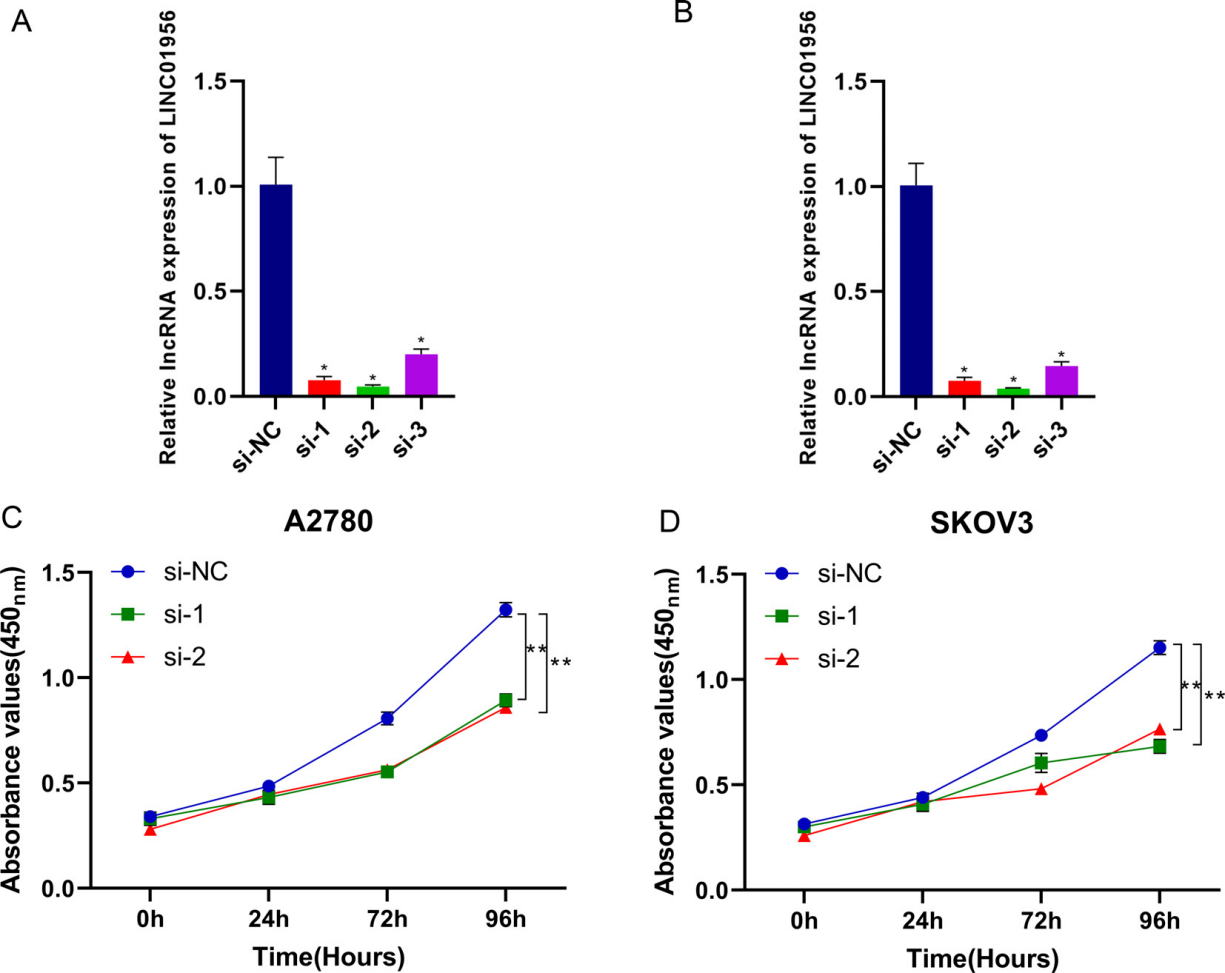
siRNA Screening and CCK8 Assay. siRNA-1, siRNA-2, and siRNA-3 were all effective in knocking down LINC01956 in A2780 [**(A)**, *P*< 0.05] and SKOV3 [**(B)**, *P<* 0.05] cells. A2780 [**(C)**, *P<* 0.01] and SKOV3 [**(D)**, *P<* 0.01] cells, with knocked-down expression of LINC01956, exhibited suppressed cell proliferation capabilities. *Indicates a p-value of 0.05, **indicates a p-value of 0.01.

Firstly, we selected three siRNAs to treat human OC cells A2780 and SKOV3, and found that siRNA1 and siRNA2 were more effective than siRNA3, hence siRNA1 and siRNA2 were used for subsequent experimental studies (**Figures 6A, B** *P*<0.05). The primer sequences of this study were detailed in **Supplementary Table 3**. In human OC cells A2780 and SKOV3, we observed a significant reduction in the proliferative capacity of both cell lines when LINC01956 was knocked down (**Figures 6C, D** *P*<0.01).

### Impact of LINC01956 knockdown on cell invasion and migration

**Figure 7:**
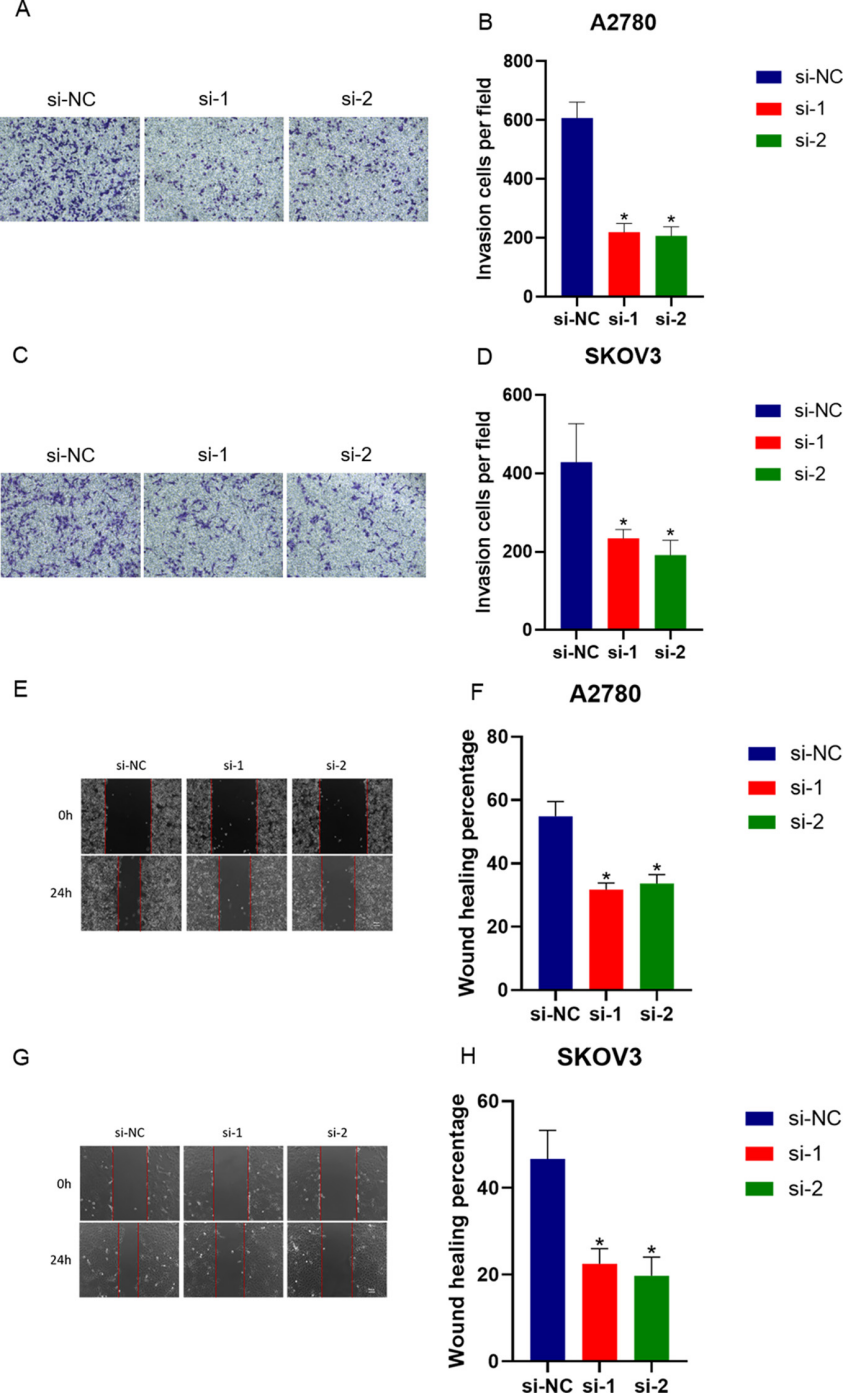
Invasion and migration assays of A2780 and SKOV3 cells. A2780 and SKOV3 cells with knocked-down expression of LINC01956 showed inhibited abilities in cell invasion [**(A–D)**, P< 0.05] and migration [**(E–H)**, P< 0.05]. * means P<0.05.

The Transwell assay revealed that the invasive capabilities of the A2780 group (**Figures 7A, B**) and the values observed in the SKOV3 group (**Figures 7C, D**) were markedly reduced compared to the blank control group, with statistical significance (*P*<0.05). The scratch test demonstrated that, 24 hours post-scratch, the migration ability, cell count, and confluence of the A2780 group (**Figures 7E, F**) and SKOV3 group (**Figures 7G, H**) with knockdown of LINC01956 were significantly reduced compared to the blank control group (P<0.05).

## Discussion

In this study, we delved into the role of CuLncs in OC, with the aim of identifying potential prognostic biomarkers and therapeutic targets. Through comprehensive analysis of the transcriptome data from 376 OC patients, we identified a set of 8 CuLncs that served as risk signatures. Our findings revealed that the high-risk group, as defined by these CuLncs, exhibited significantly poorer survival rates compared to the low-risk group. Notably, we discovered that drugs such as JQ12, PD-0325901, and sorafenib showed promising sensitivity in the high-risk group, suggesting they may serve as effective treatment options for patients with poor prognoses. In addition to these clinical insights, we conducted *in vitro* experiments to investigate the functional role of LINC01956, one of the key CuLncs identified in our risk signature. We found that the knockdown of LINC01956 significantly inhibited the proliferation, invasion, and migration of OC cells, providing a cellular basis for its role in the progression of OC.

Cuproptosis, as a novel cellular death mechanism, has become a hot topic of research in recent years (43–46). Numerous studies have indicated that genes associated with cuproptosis play a significant role in the onset and progression of cancer (47–50). For instance, Sun and colleagues utilized the TCGA-OV dataset as a training group to construct a prognostic classification system based on CRGs for predicting the prognosis of OC patients. This model was validated on the GSE26193 dataset, confirming a significant negative correlation between high-risk scores and patients’ overall survival (OS) (*P*<0.0001) (51). Additionally, research has found that risk scores constructed based on cuproptosis-related genes correlate with immune checkpoint molecules, such as PD-L1, CTLA4, targeted therapy-related genes, cancer stem cell characteristics, and sensitivity to chemotherapy and targeted drugs (52). In the field of lncRNA, CuLncs are also increasingly becoming the focus of research (19, 53). Guo and colleagues built a model to predict the survival time of OC patients based on CuLncs, but have not yet experimentally verified the associated lncRNAs *in vitro (* 54). Wang and colleagues established a prognostic model for OC using 6 CuLncs and validated the expression differences of the key lncRNA CTC.246B18.8 in OC cell lines and normal ovarian epithelial cells (55). Li and colleagues, based on the prognostic model, knocked down key lncRNAs and used the CCK8 method to assess their impact on the proliferative capacity of OC cells (56). Sorafenib is a well-recognized multikinase inhibitor that works by targeting multiple kinases involved in tumor growth and angiogenesis, thereby reducing the tumor burden. It has demonstrated efficacy in various types of cancers (57, 58). PD-0325901 is a MEK inhibitor, and its mechanism involves inhibiting the MEK pathway, which is often dysregulated in cancer cells, leading to reduced cell proliferation and survival (59). There are currently no relevant studies on JQ12 in the context of OC (ovarian cancer). Although the specific applications of these drugs in OC are still under investigation, their roles in other cancers suggest that they may be effective in treating OC, particularly when used in combination with other therapies. Building upon the work of predecessors, this study has not only constructed a prognostic model with high predictive accuracy and explored potential therapeutic drugs, but more importantly, through CCK8 assays, Transwell assays, and scratch assays, our study has validated that the suppression of LINC01956 markedly impedes the proliferative, invasive, and migratory capacities of OC cells. This discovery introduces a novel potential therapeutic target for the diagnosis and management of OC, highlighting the substantial promise of CuLncs in OC research.

It must be acknowledged that this study does have certain limitations. First, the study is confined to 376 OC patients, which is a relatively small sample size. The data are entirely derived from the TCGA-OV database, which may introduce selection bias. Second, although the study has identified several CuLncs associated with OC prognosis and validated their effects on cell proliferation, invasion, and migration *in vitro*, the *in vivo* relevance of these findings remains to be explored. Animal models or clinical trials are necessary to assess the therapeutic potential of targeting these CuLncs in a more physiologically relevant context. Third, this study focuses on the correlation between CuLncs and OC prognosis but does not delve deeply into the underlying molecular mechanisms. Future research should aim to elucidate the specific pathways through which these CuLncs influence cancer progression, including their interactions with other cellular components and their role in modulating the tumor microenvironment. Lastly, the drug sensitivity analysis identifies potential therapeutic agents for high-risk OC patients, but these findings are based on computational predictions. Further experimental validation, including *in vitro* and *in vivo* studies, is required to confirm the efficacy and safety of these drugs in treating OC.

Future research should build on the findings of this study and focus on several key areas. First, conducting validation studies in independent cohorts will help confirm the prognostic value of the identified CuLncs. Second, exploring the molecular mechanisms underlying the role of CuLncs in OC progression is crucial. Additionally, investigating the role of CuLncs in the tumor microenvironment, particularly their impact on immune cell infiltration and function, could reveal new therapeutic targets for immunotherapy. Third, further *in vivo* studies using animal models are needed to validate the therapeutic potential of targeting CuLncs. In summary, while this study provides a promising foundation for understanding the role of CuLncs in OC, further research is needed to fully realize their potential in improving the prognosis and treatment of this devastating disease.

## Conclusion

To summarize, our study successfully identified a prognostic risk model based on CuLncs in ovarian cancer, demonstrating significant predictive power and potential therapeutic applications, while highlighting the role of CuLncs in immune response and tumor progression.

## Data Availability

The original contributions presented in the study are included in the article/**Supplementary Material**. Further inquiries can be directed to the corresponding authors.

## Supplementary material

Supplementary Figure 1 Defining the CuLncs with prognostic significance in OC. A total of 248 CuLncs were identified in OC **(A)**. The forest plot revealed CuLncs with significant prognostic value **(B)**. The 10-fold cross-validation of variable selection in the least absolute shrinkage and selection operator (LASSO) algorithm **(C)**. Correlation of lncRNAs with CRGs in the prognostic signature **(D)**.

Supplementary Figure 2 Testing the Prognostic Model’s Performance The results of both univariate and multivariate analysis of independent prognostic factors were analyzed to determine the risk scores for OS **(A, B)**. A nomogram considering clinicopathological variables and risk scores predicts OS in OC **(C)**.

Supplementary Figure 3 Immune Landscape of OC Patients in High and Low Risk Groups The Tumor Immune Dysfunction and Exclusion (TIDE) scores revealed that the high-risk OC group exhibited significantly higher TIDE scores **(A)**. The heatmap of immune activity indicated that the high-risk OC group had higher expression levels of gene sets associated with MHC I expression (p< 0.001), Type I interferon (INF) response, pro-inflammatory activity, and cytolytic activity (p< 0.01) compared to other immune-related pathways (p< 0.05) **(B)**.

## References

[1] Du P Xu X Wang Y. Hsa_circ_0000585 promotes chemoresistance to cis-platin in epithelial cells of ovarian cancer by modulating autophagy. Biochem Biophys Res Commun. (2023) 678:186–92. doi: 10.1016/j.bbrc.2023.08.048 37643536

[2] Liu G Feng Y Li J Deng T Yin A Yan L. A novel combination of niraparib and anlotinib in platinum-resistant ovarian cancer: Efficacy and safety results from the phase II, multi-center ANNIE study. EClinicalMedicine. (2022) 54:101767. doi: 10.1016/j.eclinm.2022.101767 36583171 PMC9793276

[3] Webb PM Jordan SJ. Global epidemiology of epithelial ovarian cancer. Nat Rev Clin Oncol. (2024) 21:389–400. doi: 10.1038/s41571-024-00881-3 38548868

[4] Kahn RM Gordhandas S Godwin K Stone RL Worley MJ Jr Lu KH. Salpingectomy for the primary prevention of ovarian cancer: A systematic review. JAMA Surg. (2023) 158:1204–11. doi: 10.1001/jamasurg.2023.4164 PMC11185162 37672283

[5] Siegel RL Miller KD Wagle NS Jemal A. Cancer statistics, 2023. CA Cancer J Clin. (2023) 73:17–48. doi: 10.3322/caac.21763 36633525

[6] He S Wang W Wan Z Shen H Zhao Y You Z. FAM83B inhibits ovarian cancer cisplatin resistance through inhibiting Wnt pathway. Oncogenesis. (2021) 10:6. doi: 10.1038/s41389-020-00301-y 33423038 PMC7797002

[7] Lheureux S Gourley C Vergote I Oza AM. Epithelial ovarian cancer. Lancet. (2019) 393:1240–53. doi: 10.1016/S0140-6736(18)32552-2 30910306

[8] Sideris M Menon U Manchanda R. Screening and prevention of ovarian cancer. Med J Aust. (2024) 220:264–74. doi: 10.5694/mja2.v220.5 PMC7617385 38353066

[9] Wang S Liu Y Xiao H Chen Z Yang X Yin J. Inhibition of SF3B1 improves the immune microenvironment through pyroptosis and synergizes with αPDL1 in ovarian cancer. Cell Death Dis. (2023) 14:775. doi: 10.1038/s41419-023-06301-1 38012150 PMC10682409

[10] Steinbuch SC Lüß AM Eltrop S Götte M Kiesel L. Endometriosis-associated ovarian cancer: from molecular pathologies to clinical relevance. Int J Mol Sci. (2024) 25. doi: 10.3390/ijms25084306 PMC11050613 38673891

[11] Launonen IM Vähärautio A Färkkilä A. The emerging role of the single-cell and spatial tumor microenvironment in high-grade serous ovarian cancer. Cold Spring Harb Perspect Med. (2023) 13. doi: 10.1101/cshperspect.a041314 PMC10547388 37553211

[12] Tang D Chen X Kroemer G. Cuproptosis: a copper-triggered modality of mitochondrial cell death. Cell Res. (2022) 32:417–8. doi: 10.1038/s41422-022-00653-7 PMC9061796 35354936

[13] Wang Y Zhang L Zhou F. Cuproptosis: a new form of programmed cell death. Cell Mol Immunol. (2022) 19:867–8. doi: 10.1038/s41423-022-00866-1 PMC9338229 35459854

[14] Xu M Mu J Wang J Zhou Q Wang J. Construction and validation of a cuproptosis-related lncRNA signature as a novel and robust prognostic model for colon adenocarcinoma. Front Oncol. (2022) 12:961213. doi: 10.3389/fonc.2022.961213 35965536 PMC9367690

[15] Lu D Liao J Cheng H Ma Q Wu F. Construction and systematic evaluation of a machine learning-based cuproptosis-related lncRNA score signature to predict the response to immunotherapy in hepatocellular carcinoma. Front Immunol. (2023) 14:1097075. doi: 10.3389/fimmu.2023.1097075 36761763 PMC9905126

[16] Wang L Li Y Wang Y Li J Sun Y. Identification of cuproptosis-related lncRNAs for prognosis and immunotherapy in glioma. J Cell Mol Med. (2022) 26:5820–31. doi: 10.1111/jcmm.v26.23 PMC9716210 36317420

[17] Bai Y Zhang Q Liu F Quan J. A novel cuproptosis-related lncRNA signature predicts the prognosis and immune landscape in bladder cancer. Front Immunol. (2022) 13:1027449. doi: 10.3389/fimmu.2022.1027449 36451815 PMC9701814

[18] Xie M Cheng B Yu S He Y Cao Y Zhou T. Cuproptosis-related miR-21-5p/FDX1 axis in clear cell renal cell carcinoma and its potential impact on tumor microenvironment. Cells. (2022) 12. doi: 10.3390/cells12010173 PMC9818076 36611966

[19] Liu L Wang Q Zhou JY Zhang B. Developing four cuproptosis-related lncRNAs signature to predict prognosis a nd immune activity in ovarian cancer. J Ovarian Res. (2023) 16:88. doi: 10.1186/s13048-023-01165-7 37122030 PMC10150549

[20] Huang EM Ma N Ma T Zhou JY Yang WS Liu CX. Cuproptosis-related long non-coding RNAs model that effectively predicts prognosis in hepatocellular carcinoma. World J Gastrointest Oncol. (2022) 14:1981–2003. doi: 10.4251/wjgo.v14.i10.1981 36310708 PMC9611437

[21] Ma S Zhu J Wang M Zhu J Wang W Xiong Y. A cuproptosis-related long non-coding RNA signature to predict the prognosis and immune microenvironment characterization for lung adenocarcinoma. Transl Lung Cancer Res. (2022) 11:2079–93. doi: 10.21037/tlcr-22-660 PMC9641048 36386454

[22] Ma C Li F Gu Z Yang Y Qi Y. A novel defined risk signature of cuproptosis-related long non-coding RNA for predicting prognosis, immune infiltration, and immunotherapy response in lung adenocarcinoma. Front Pharmacol. (2023) 14:1146840. doi: 10.3389/fphar.2023.1146840 37670938 PMC10475834

[23] Jiang ZR Yang LH Jin LZ Yi LM Bing PP. Identification of novel cuproptosis-related lncRNA signatures to predict the prognosis and immune microenvironment of breast cancer patients. Front Oncol. (2022) 12:988680. doi: 10.3389/fonc.2022.988680 36203428 PMC9531154

[24] Wu C Tan J Wang X Qin C Long W Pan Y. Pan-cancer analyses reveal molecular and clinical characteristics of cuproptosis regulators. Imeta. (2023) 2:e68. doi: 10.1002/imt2.v2.1 38868340 PMC10989956

[25] Chen Z Liu J Zheng M Mo M Hu X Liu C. TRIM24-DTNBP1-ATP7A mediated astrocyte cuproptosis in cognition and memory dysfunction caused by Y(2)O(3) NPs. Sci Total Environ. (2024) 954:176353. doi: 10.1016/j.scitotenv.2024.176353 39304169

[26] Sun Z Zhao Q Zhang J Hu Y Qu J. Bioinformatics reveals diagnostic potential of cuproptosis-related genes in the pathogenesis of sepsis. Heliyon. (2024) 10:e22664. doi: 10.1016/j.heliyon.2023.e22664 38163157 PMC10754710

[27] Liu WQ Lin WR Yan L Xu WH Yang J. Copper homeostasis and cuproptosis in cancer immunity and therapy. Immunol Rev. (2024) 321:211–27. doi: 10.1111/imr.v321.1 37715546

[28] Wang W Li S Huang Y Guo J Sun L Sun G. Comprehensive analysis of the potential biological significance of cuproptosis-related gene LIPT2 in pan-cancer prognosis and immunotherapy. Sci Rep. (2023) 13:22910. doi: 10.1038/s41598-023-50039-x 38129565 PMC10739704

[29] Lai SW Weng PW Yadav VK Pikatan NW Yeh CT Hsieh MS. Underlying mechanisms of novel cuproptosis-related dihydrolipoamide branched-chain transacylase E2 (DBT) signature in sunitinib-resistant clear-cell renal cell carcinoma. Aging (Albany NY). (2024) 16:2679–701. doi: 10.18632/aging.205504 PMC10911363 38305803

[30] Liu J Tang H Chen F Li C Xie Y Kang R. NFE2L2 and SLC25A39 drive cuproptosis resistance through GSH metabolism. Sci Rep. (2024) 14:29579. doi: 10.1038/s41598-024-81317-x 39609608 PMC11605005

[31] Li P Sun Q Bai S Wang H Zhao L. Combination of the cuproptosis inducer disulfiram and anti−PD−L1 abolishes NSCLC resistance by ATP7B to regulate the HIF−1 signaling pathway. Int J Mol Med. (2024) 53. doi: 10.3892/ijmm.2024.5446 PMC10781418 38186308

[32] Qiao L Zhu G Jiang T Qian Y Sun Q Zhao G. Self-destructive copper carriers induce pyroptosis and cuproptosis for efficient tumor immunotherapy against dormant and recurrent tumors. Adv Mater. (2024) 36:e2308241. doi: 10.1002/adma.202308241 37820717

[33] Cheng X Yang F Li Y Cao Y Zhang M Ji J. The crosstalk role of CDKN2A between tumor progression and cuproptosis resistance in colorectal cancer. Aging (Albany NY). (2024) 16:10512–38. doi: 10.18632/aging.205945 PMC11236303 38888512

[34] Ma H Ge Y Li Y Wang T Chen W. Construction of a prognostic model based on cuproptosis-related genes and exploration of the value of DLAT and DLST in the metastasis for non-small cell lung cancer. Med (Baltimore). (2024) 103:e40727. doi: 10.1097/MD.0000000000040727 PMC11631004 39654205

[35] Liao Y Wang D Gu C Wang X Zhu S. A cuproptosis nanocapsule for cancer radiotherapy. Nat Nanotechnol. (2024) 19:1892–902. doi: 10.1038/s41565-024-01784-1 39300223

[36] Wang C Guo J Zhang Y Zhou S Jiang B. Cuproptosis-related gene FDX1 suppresses the growth and progression of colorectal cancer by retarding EMT progress. Biochem Genet. (2024). doi: 10.1007/s10528-024-10784-8 PMC11832605 38520567

[37] Deng R Zhu L Jiang J Chen J Li H. Cuproptosis-related gene LIPT1 as a prognostic indicator in non-small cell lung cancer: Functional involvement and regulation of ATOX1 expression. Biomol BioMed. (2024) 24:647–58. doi: 10.17305/bb.2023.9931 PMC11088889 38041690

[38] Li X Rui J Yang Z Shang-Guan F Shi H Wang D. Cuproptosis related gene DLD associated with poor prognosis and Malignant biological characteristics in lung adenocarcinoma. Curr Cancer Drug Targets. (2024) 24:867–80. doi: 10.2174/0115680096271679231213060750 38310466

[39] Guo P Niu Z Zhang D Zhao F Li J Lu T. Potential impact of cuproptosis-related genes on tumor immunity in esophageal carcinoma. Aging (Albany NY). (2023) 15:15535–56. doi: 10.18632/aging.205391 PMC10781504 38159255

[40] He R Li Y Jiao P Huang Y Dong S. Cuproptosis-related genes score and its hub gene GCSH: A novel predictor for cholangiocarcinomas prognosis based on RNA seq and experimental analyses. J Cancer. (2024) 15:1551–67. doi: 10.7150/jca.92327 PMC10869970 38370386

[41] Hu Q Zhang X Huang J Peng H Sun Y Sang W. The STAT1-SLC31A1 axis: Potential regulation of cuproptosis in diabetic retinopathy. Gene. (2024) 930:148861. doi: 10.1016/j.gene.2024.148861 39153705

[42] Zhang C Wang S Tang H Lai R Cai Q Su Y. Prognostic and immunological role of cuproptosis-related gene MTF1 in pan-cancer. J Cancer. (2024) 15:5786–809. doi: 10.7150/jca.98749 PMC11414622 39308676

[43] Huo S Wang Q Shi W Peng L Jiang Y Zhu M. ATF3/SPI1/SLC31A1 signaling promotes cuproptosis induced by advanced glycosylation end products in diabetic myocardial injury. Int J Mol Sci. (2023) 24. doi: 10.3390/ijms24021667 PMC9862315 36675183

[44] Sun L Zhang Y Yang B Sun S Zhang P Luo Z. Lactylation of METTL16 promotes cuproptosis via m(6)A-modification on FDX1 mRNA in gastric cancer. Nat Commun. (2023) 14:6523. doi: 10.1038/s41467-023-42025-8 37863889 PMC10589265

[45] Yang W Wang Y Huang Y Yu J Wang T Li C. 4-Octyl itaconate inhibits aerobic glycolysis by targeting GAPDH to promote cuproptosis in colorectal cancer. BioMed Pharmacother. (2023) 159:114301. doi: 10.1016/j.biopha.2023.114301 36706634

[46] Chen Y Li X Sun R Ji J Yang F Tian W. A broad cuproptosis landscape in inflammatory bowel disease. Front Immunol. (2022) 13:1031539. doi: 10.3389/fimmu.2022.1031539 36405733 PMC9669451

[47] Nie X Chen H Xiong Y Chen J Liu T. Anisomycin has a potential toxicity of promoting cuproptosis in human ovarian cancer stem cells by attenuating YY1/lipoic acid pathway activation. J Cancer. (2022) 13:3503–14. doi: 10.7150/jca.77445 PMC9723990 36484005

[48] Gan Y Liu T Feng W Wang L Li LI. Drug repositioning of disulfiram induces endometrioid epithelial ovarian cancer cell death via the both apoptosis and cuproptosis pathways. Oncol Res. (2023) 31:333–43. doi: 10.32604/or.2023.028694 PMC10229305 37305383

[49] Song Q Zhou R Shu F Fu W. Cuproptosis scoring system to predict the clinical outcome and immune response in bladder cancer. Front Immunol. (2022) 13:958368. doi: 10.3389/fimmu.2022.958368 35990642 PMC9386055

[50] Liu X Luo B Wu X Tang Z. Cuproptosis and cuproptosis-related genes: Emerging potential therapeutic targets in breast cancer. Biochim Biophys Acta Rev Cancer. (2023) 1878:189013. doi: 10.1016/j.bbcan.2023.189013 37918452

[51] Sun X Xu P Zhang F Sun T Jiang H Lu X. The cuproptosis-related gene signature serves as a potential prognostic predictor for ovarian cancer using bioinformatics analysis. Ann Transl Med. (2022) 10:1021. doi: 10.21037/atm-22-4546 36267774 PMC9577750

[52] Zhang J Lu M Xu H Ren F Zhu L. Molecular subtypes based on cuproptosis-related genes and tumor microenvironment infiltration characterization in ovarian cancer. Cancer Cell Int. (2022) 22:328. doi: 10.1186/s12935-022-02756-y 36307842 PMC9617300

[53] Chen R Huang Y Sun K Dong F Wang X Guan J. Construction of a prognostic model for ovarian cancer based on a comprehensive bioinformatics analysis of cuproptosis-associated long non-coding RNA signatures. Heliyon. (2024) 10:e35004. doi: 10.1016/j.heliyon.2024.e35004 39170367 PMC11336372

[54] Guo J Zhou M Li J Yang Y Hu Y. The prognosis and immunotherapy prediction model of ovarian serous cystadenocarcinoma patient was constructed based on cuproptosis-related lncRNA. Tohoku J Exp Med. (2024) 262:63–74. doi: 10.1620/tjem.2023.J056 37438122

[55] Wang Y Liang Q Xu L Xiong J Gao K. Cuproptosis-related lncRNAs ovarian cancer: Multi-omics analysis of molecular mechanisms and potential therapeutic targets. Environ Toxicol. (2024) 39:1650–65. doi: 10.1002/tox.24067 38019212

[56] Li N Yu K Huang D Li S Zeng D. Molecular characterization of cuproptosis-related lncRNAs: defining molecular subtypes and a prognostic signature of ovarian cancer. Biol Trace Elem Res. (2024) 202:1428–45. doi: 10.1007/s12011-023-03780-3 37528285

[57] Leone Roberti Maggiore U Menada Valenzano M Venturini PL Ferrero S. Sorafenib for ovarian cancer. Expert Opin Investig Drugs. (2013) 22:1049–62. doi: 10.1517/13543784.2013.802769 23675696

[58] Tian C Liu Y Xue L Zhang D Zhang X Su J. Sorafenib inhibits ovarian cancer cell proliferation and mobility and induces radiosensitivity by targeting the tumor cell epithelial-mesenchymal transition. Open Life Sci. (2022) 17:616–25. doi: 10.1515/biol-2022-0066 PMC9202537 35800071

[59] Wainberg ZA Alsina M Soares HP Braña I Britten CD Conte Del G. A multi-arm phase I study of the PI3K/mTOR inhibitors PF-04691502 and gedatolisib (PF-05212384) plus irinotecan or the MEK inhibitor PD-0325901 in advanced cancer. Target Oncol. (2017) 12:775–85. doi: 10.1007/s11523-017-0530-5 PMC5700209 29067643

