# Supplementary figures and images for: Big data analysis and machine learning of the role of cuproptosis-related long non-coding RNAs (CuLncs) in the prognosis and immune landscape of ovarian cancer
Source: Front Immunol. 2025 Feb 25;16:1555782. doi: 10.3389/fimmu.2025.1555782 (PMC11893572; doi:10.3389/fimmu.2025.1555782)

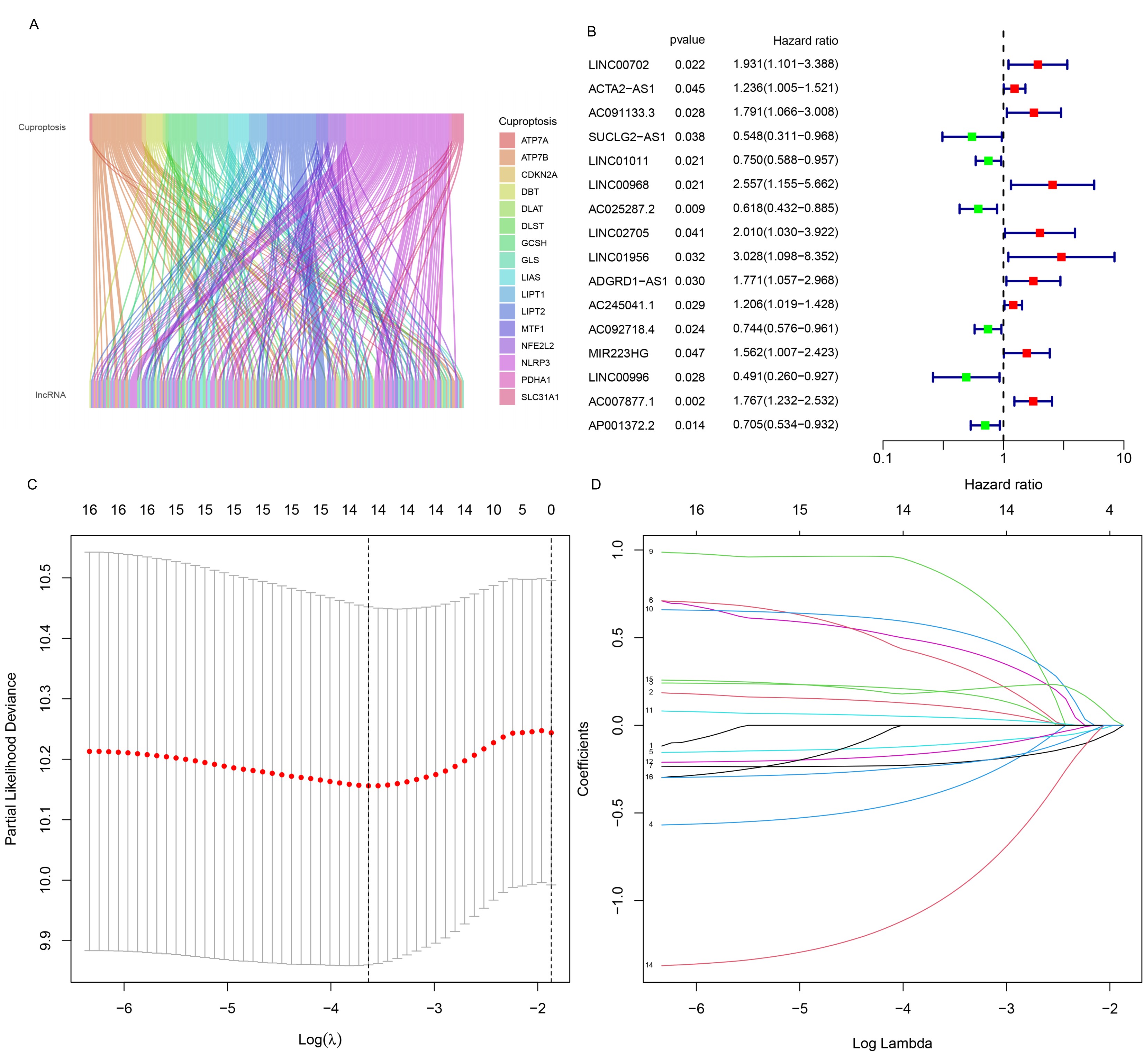

Supplement: Supplementary Figure 1 — Defining the CuLncs with prognostic significance in OC. A total of 248 CuLncs were identified in OC (A). The forest plot revealed CuLncs with significant prognostic value (B). The 10-fold cross-validation of variable selection in the least absolute shrinkage and selection operator (LASSO) algorithm (C). Correlation of lncRNAs with CRGs in the prognostic signature (D). [file Image1.jpeg]

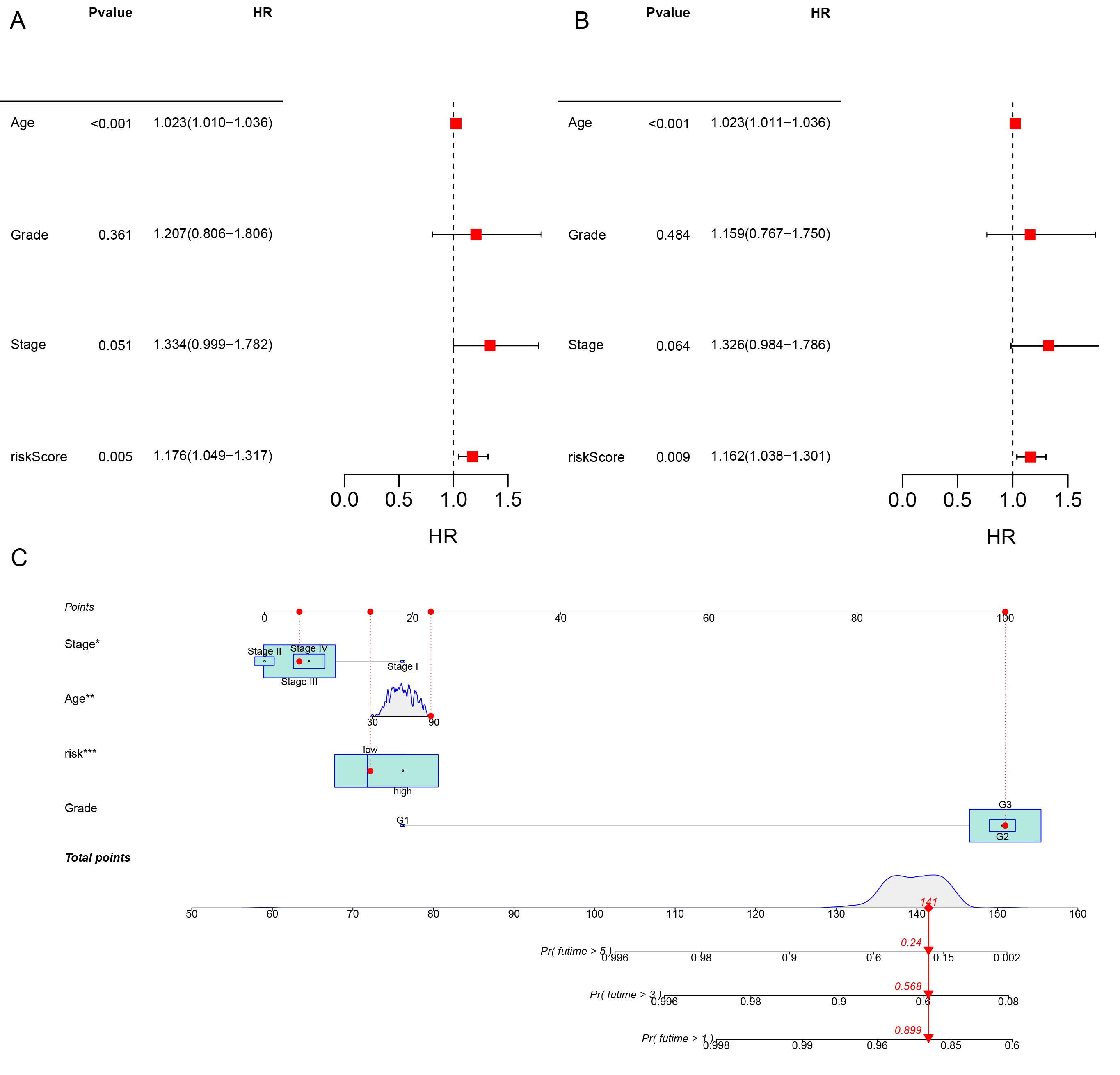

Supplement: Supplementary Figure 2 — Testing the Prognostic Model’s Performance The results of both univariate and multivariate analysis of independent prognostic factors were analyzed to determine the risk scores for OS (A, B). A nomogram considering clinicopathological variables and risk scores predicts OS in OC (C). [file Image2.jpeg]

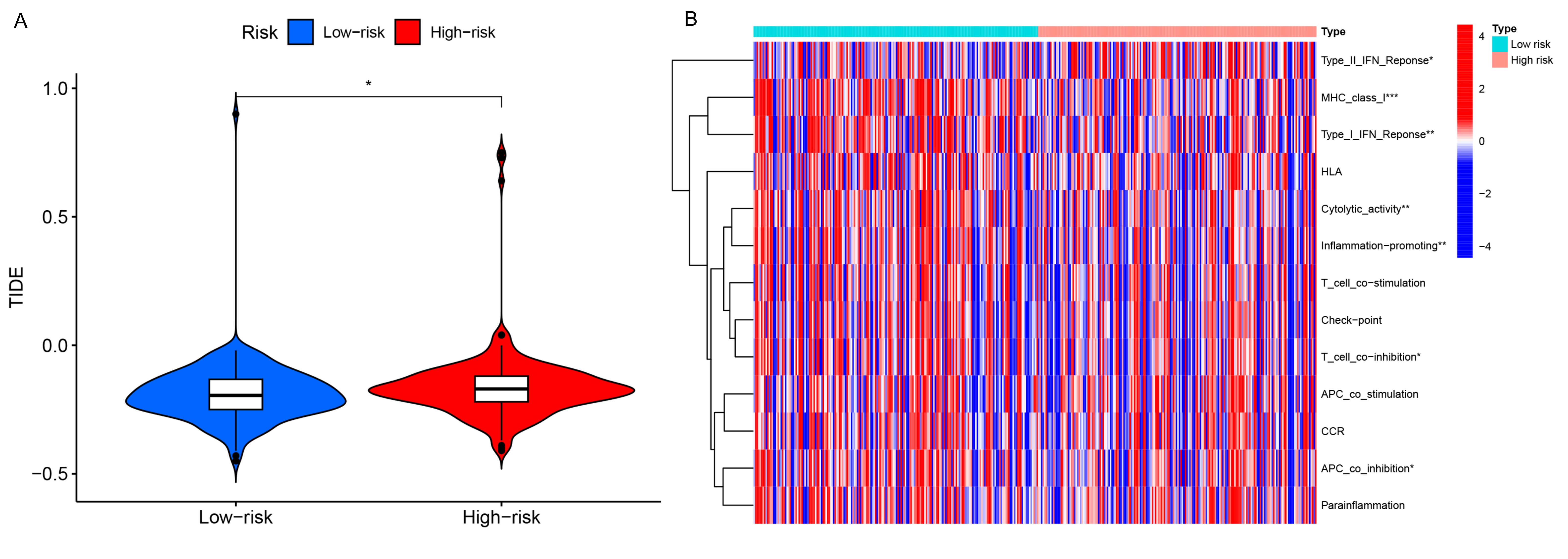

Supplement: Supplementary Figure 3 — Immune Landscape of OC Patients in High and Low Risk Groups The Tumor Immune Dysfunction and Exclusion (TIDE) scores revealed that the high-risk OC group exhibited significantly higher TIDE scores (A). The heatmap of immune activity indicated that the high-risk OC group had higher expression levels of gene sets associated with MHC I expression (p< 0.001), Type I interferon (INF) response, pro-inflammatory activity, and cytolytic activity (p< 0.01) compared to other immune-related pathways (p< 0.05) (B). [file Image3.jpeg]
