# Supplementary material for: Big data analysis and machine learning of the role of cuproptosis-related long non-coding RNAs (CuLncs) in the prognosis and immune landscape of ovarian cancer
Source: Front Immunol. 2025 Feb 25;16:1555782. doi: 10.3389/fimmu.2025.1555782 (PMC11893572; doi:10.3389/fimmu.2025.1555782)
Supplement: Supplementary file 4 [file Table1.docx]

Table 1

Clinical information statistics of training set and test set.

| Characteristics | Type | Total | Training | Test | *P* value |
| --- | --- | --- | --- | --- | --- |
| Age | <=65 | 258(68.62%) | 132(70.21%) | 126(67.02%) | 0.5784 |
|  | >65 | 118(31.38%) | 56(29.79%) | 62(32.98%) |  |
| Grade | G1 | 1(0.27%) | 1(0.53%) | 0(0%) | 0.3975 |
|  | G2 | 42(11.17%) | 18(9.57%) | 24(12.77%) |  |
|  | G3 | 322(85.64%) | 162(86.17%) | 160(85.11%) |  |
|  | Unknown | 11(2.93%) | 7(3.72%) | 4(2.13%) |  |
| Stage | Stage I | 1(0.27%) | 0(0%) | 1(0.53%) | 0.5898 |
|  | Stage II | 22(5.85%) | 9(4.79%) | 13(6.91%) |  |
|  | Stage III | 292(77.66%) | 149(79.26%) | 143(76.06%) |  |
|  | Stage IV | 58(15.43%) | 28(14.89%) | 30(15.96%) |  |
|  | Unknown | 3(0.8%) | 2(1.06%) | 1(0.53%) |  |

Notes: The p values for differences in clinical characteristics between the two sets according to the chi-square test were greater than 0.05; that is, there were no significant differences in clinical features between the groups.
