# Supplementary material for: Big data analysis and machine learning of the role of cuproptosis-related long non-coding RNAs (CuLncs) in the prognosis and immune landscape of ovarian cancer
Source: Front Immunol. 2025 Feb 25;16:1555782. doi: 10.3389/fimmu.2025.1555782 (PMC11893572; doi:10.3389/fimmu.2025.1555782)
Supplement: Supplementary file 5 [file Table2.docx]

Table 2

Eight cuproptosis-related lncRNAs identified by multivariate Cox regression analysis.

| ID | coef | HR | HR.95L | HR.95H | P value |
| --- | --- | --- | --- | --- | --- |
| SUCLG2-AS1 | -0.662014443 | 1.236353069 | 1.004797922 | 1.521269977 | 0.044941545 |
| LINC00968 | 1.035582271 | 2.557335702 | 1.155048599 | 5.662069888 | 0.020590705 |
| AC025287.2 | -0.469684948 | 0.618347452 | 0.431855346 | 0.885374176 | 0.008672383 |
| LINC02705 | 0.744272833 | 2.010054117 | 1.030247076 | 3.921697668 | 0.040623922 |
| LINC01956 | 1.377998861 | 3.02806028 | 1.097898832 | 8.35154278 | 0.03232242 |
| ADGRD1-AS1 | 0.683869099 | 1.770772572 | 1.056607899 | 2.967643441 | 0.030084652 |
| LINC00996 | -1.36584804 | 0.490633047 | 0.259697 | 0.926929409 | 0.02825478 |
| AP001372.2 | -0.299357825 | 0.705420937 | 0.534010926 | 0.931851156 | 0.01401385 |

Notes: Coef, coefficient; HR, hazard ratio.
