# Supplementary material for: Big data analysis and machine learning of the role of cuproptosis-related long non-coding RNAs (CuLncs) in the prognosis and immune landscape of ovarian cancer
Source: Front Immunol. 2025 Feb 25;16:1555782. doi: 10.3389/fimmu.2025.1555782 (PMC11893572; doi:10.3389/fimmu.2025.1555782)
Supplement: Supplementary file 6 [file Table3.docx]

Table 3

The sequences of primers used in the qRT‒PCR experiment.

| Primer name | Forward (5'→3') | Reverse (3'→5') |
| --- | --- | --- |
| LINC01956 | GCCACGTTCATTGCACAGTT | TCACTTTGCACCACAATGCG |
| Si 1 | UGGAUUUGUACCAUUCUUCUG | GAAGAAUGGUACAAAUCCAAG |
| Si 2 | UUUUGAGUGGGUAUCAACCAG | GGUUGAUACCCACUCAAAAAG |
| Si 3 | AGUUUCAACCGUCUUAAUCAG | GAUUAAGACGGUUGAAACUAG |
| GAPDH | AATGACCCCTTCATTGAC | TCCACGACGTACTCAGCGC |
